# Pomegranate Peel Powder as a Food Preservative in Fruit Salad: A Sustainable Approach

**DOI:** 10.3390/foods10061359

**Published:** 2021-06-11

**Authors:** Valentina Lacivita, Anna Lucia Incoronato, Amalia Conte, Matteo Alessandro Del Nobile

**Affiliations:** Department of Agricultural Sciences, Food and Environment, University of Foggia, Via Napoli, 25-71121 Foggia, Italy; valentina.lacivita@unifg.it (V.L.); annalucia.incoronato@unifg.it (A.L.I.); matteo.delnobile@unifg.it (M.A.D.N.)

**Keywords:** fresh-cut fruit, pomegranate peel powder, natural preservative, by-product, sustainable approach

## Abstract

This study aimed to assess the potential of pomegranate peel powder as a natural preservative. Its effects were tested on fruit salad quality decay during refrigerated storage. Nectarine and pineapple, equally portioned in polypropylene containers and covered with fructose syrup, were closed using a screw cap in air, with and without the addition of a by-product peel powder. Specifically, amounts of 2.5% and 5% (*w*/*v*) of pomegranate peel powder were put into each container. Both the microbiological and sensory qualities of the fruit salad were monitored during storage at 5 °C for 28 days. The results demonstrated that the fruit salad with the by-products showed lower counts of total mesophilic bacteria, total psychrotrophic microorganisms, yeasts, and lactic acid bacteria compared to the control, thus confirming the recognized antimicrobial properties of pomegranate peel. The other interesting finding of this study is that the addition of the investigated by-product in fruit salad did not worsen the main sensory attributes of fresh-cut fruit. Therefore, these preliminary results suggest that pomegranate peel powder has potential applications as a natural preservative in the fresh-cut food sector.

## 1. Introduction

Fruit intake, which is associated with a correct and healthy diet, is increasingly widespread among consumers. Fruit is rich in carbohydrates, minerals, amino acids, vitamins, and other nutrients that bring various benefits to human health [1]. In the last few years, fresh-cut fruit has been popular because it meets consumers’ need for fresh, natural, and convenient food. Various formats are available in the refrigerated section, ranging from single fruit to fruit salads. However, these fresh-cut products are all highly perishable [2]. Minimal processing operations, which include peeling, slicing, dicing, etc., can cause damage to fruits’ surfaces, thus limiting their shelf life compared to unprocessed whole fruits [3,4,5]. Damage caused by the cutting process typically occurs in the form of tissue softening, water loss, color change (surface browning), microbial proliferation, and the appearance of unpleasant odors [4,5,6,7]. In general, the most common preservation systems for fresh-cut fruit include cold storage, the use of modified atmosphere packaging, coating application, or the addition of synthetic preservatives [8,9,10]. However, consumers are increasingly inclined to purchase fresh-cut fruit without synthetic additives, as they have a greater awareness of health and food safety. An alternative to synthetic additives could be the use of preservatives of natural origin, such as essential oils, enzymes, and organic acids [11], or lactic acid bacteria and derived bacteriocins [12].

In this context, by-products from fruit and vegetable processing offer an interesting alternative. They are good sources of bioactive compounds and well-recognized for their relevant antimicrobial and antioxidant properties [13,14].

Among fruit and vegetable by-products, pomegranate peel makes up about 40–50% of total fruit weight. This by-product is one of the most abundant wastes discarded during juice, jam, and jelly production [15]. Pomegranate peel contains greater amounts of flavonoids, phenolic acids, tannins, and other compounds than other parts of the fruit [16]. These bioactive compounds have shown important health benefits, including antioxidant and anti-cancer properties [17,18] and remarkable antimicrobial activity against pathogenic and spoilage bacteria [19,20,21,22,23]. Various recent applications have shown how pomegranate peel extract can be used to develop active coatings or bio-based films to be applied to fresh food to control microbial proliferation or oxidation phenomena [24,25,26]. A few studies have reported the application of peel powder loaded in polymeric matrixes as a potential active food packaging [27,28,29]. However, the literature shows fewer examples dealing with the direct utilization of pomegranate peel powder for food preservation. In this regard, two studies can be cited. The first one is by Incoronato et al. [30] and deals with the development of new pancakes where both the juice and by-products of pomegranate were used as ingredients. Thus, the addition of by-products to the pancake formulation not only increased its nutritional content but also promoted the extension of its shelf life. Another successful example of pomegranate peel utilization was proposed by Panza et al. [31], who studied how to use pomegranate peel powder as a breading for cod sticks. This research also demonstrated that the use of pomegranate by-products could be a sustainable way to reduce the environmental impacts and costs associated with by-product disposal, with the added advantages of great product quality and increased shelf life.

To raise awareness about complete by-product recycling, their potential applications to food need to be better investigated [32]. Therefore, this study aims to support new advances in the application of pomegranate peel to fresh-cut fruit salad. For the study, the effectiveness of peel powder, at two different concentrations, on the quality decay of a mix of fresh-cut nectarine and pineapple in fructose syrup stored at 4 °C was assessed for 4 weeks. Both the microbiological and sensory qualities were investigated to demonstrate the efficacy of peel by-products on salad quality.

## 2. Materials and Methods 

### 2.1. Raw Materials and Pomegranate Peel Powder

The pomegranates (*Punica granatum*, cv. Wonderful) were kindly provided by a local horticultural association (A.P.O. Foggia, Italy). The fruits were washed in tap water to remove dust and impurities then dipped for 1 min in chlorinated water (20 mL/L), rinsed to remove chlorine residues, and air dried. The various parts of the fruit were separated manually (peel and arils). The pomegranate peel was cut into small pieces using a sharp knife and dried at 38 °C for 48 h in a dryer (Melchioni-Babele, Milan, Italy). The dried pomegranate peel was finely ground using a laboratory blender, then sieved to obtain a fine powder (500 μm), which was stored in plastic bags at 4 °C and protected from light until its use.

### 2.2. Fruit Salad Preparation

The nectarine (*Prunus persica*) and pineapple (*Ananas sativus*) were purchased in a local market (Foggia, Italy). The fruits were washed for 1 min in chlorinated water (20 mL/L), rinsed in tap water to remove chlorine residues, and then air dried. The fruits were manually peeled and cut into cubes (1 × 1 cm^2^) with a sharp knife. The freshly cut fruits were equally portioned (50 g) into 100 mL polypropylene containers, covered with 70 mL of 25% fructose syrup, then closed using a screw cap in air. Before portioning the fruit and syrup, 2.5% and 5% (*w*/*v*) of pomegranate peel powder were placed at the bottom of each container for the two active samples. These concentrations were found to be the most effective in preliminary analyses carried out by in vitro tests on generic foodborne microorganisms (two species of *Pseudomonas* spp. isolated from spoiled food, identified as *P. fluorescens* and *P. putida*). The control sample (Ctrl) consists of sole fruit salad and fructose syrup without any peel powder added. All of the samples were stored at 5 °C for 28 days. The microbiological and sensory qualities as well as pH were monitored during storage.

### 2.3. Microbiological Analysis

Under sterile conditions, 20 g of fruit salad was homogenized with a saline solution (0.9% NaCl) (Sigma, Milan, Italy). Decimal dilutions of the homogenate sample were made using the same diluent and plated on selective media to determine the specific microbial groups. Lactic acid bacteria were plated into de Man Rogosa and Sharpe (MRS) agar supplemented with cycloheximide (0.17 g/L) (Sigma, Milan, Italy) and incubated under anaerobic conditions at 37 °C for 48 h. Plate Count Agar (PCA) was used to enumerate the total mesophilic bacterial count incubated at 30 °C for 48 h, and the total psychrotrophic bacteria were incubated for 10 days at 4 °C. Yeasts and molds were determined in Sabouraud Dextrose Agar (SAB), supplemented with chloramphenicol (0.1 g/L) with incubation at 25 °C for 48 h and 5 days, respectively. Violet Red Bile Glucose Agar (VRBGA) incubated at 37 °C for 24 h was instead used for *Enterobacteriaceae*. All of the cultured media and supplements were obtained from Oxoid (Milan, Italy). The analyses were performed in duplicate on different samples, and the results were expressed as log colony-forming units/gram of fruit salad (CFU/g).

### 2.4. pH Determination

The pH levels of both the homogenized fruit salad and fructose syrup were measured. The measure was performed twice on two different samples by using a pH-meter after the appropriate calibration (Crison, Barcelona, Spain).

### 2.5. Sensory Evaluation

Seven trained judges, researchers from the University of Foggia, evaluated the sensory quality of the different fruit salad samples. They were already familiar with fresh-cut fruit before this study. However, a brief training section was also carried out to define the sensory attributes to be considered. According to the approach also available in the literature, odor, appearance, flavor, and texture were selected as sensory parameters, and a scale ranging from 1 to 5 (1 = dislike extremely; 2 = dislike moderately; 3 = neither like or dislike; 4 = like moderately; and 5 = like extremely) was used for the evaluation [33]. During the sensory analysis, the control and active fruit salads were differently coded and presented in random order to the panelists. Individually, they expressed their degree of appreciation for each attribute, and finally, using the same scale, they were also asked to judge the overall quality of each salad sample.

### 2.6. Statistical Analysis

Tests were carried out on duplicate batches. Experimental data are the average of two replicates. The results are presented as mean ± Standard Deviation (SD) and graphically reported. A statistical significance was determined by a one-way analysis of variance (ANOVA). Duncan’s multiple range test, with the option of homogeneous groups (*p* ≤ 0.05), was performed to determine significant differences among fruit salad samples. For this, STATISTICA 7.1 for Windows (StatSoft, Inc, Tulsa, OK, USA) was used.

## 3. Results and Discussion

### 3.1. Microbial Quality Decay during Refrigerated Storage

Fresh-cut fruit has a high susceptibility to microbial spoilage. High levels of carbohydrates and water and low pH values make the environment optimal for the growth of mesophilic, psychrotrophic, and lactic acid bacteria, in addition to yeasts and molds [12,34]. Therefore, to assess the effect of different concentrations of pomegranate peel powder (2.5 and 5%) on the microbial quality of fruit salad, the viable cell concentration of the main spoilage groups was monitored. The two percentages of peel used in this study were chosen based on preliminary in vitro antimicrobial tests (data not shown). As reported in the M&M Section 2, the evolution of microbial growth in fruit salad stored under refrigerated conditions (5 °C) was monitored for 28 days. During the storage period, statistically significant differences were observed between fruit salad with and without pomegranate peel powder, with the active samples being less contaminated.

**Figure 1:**
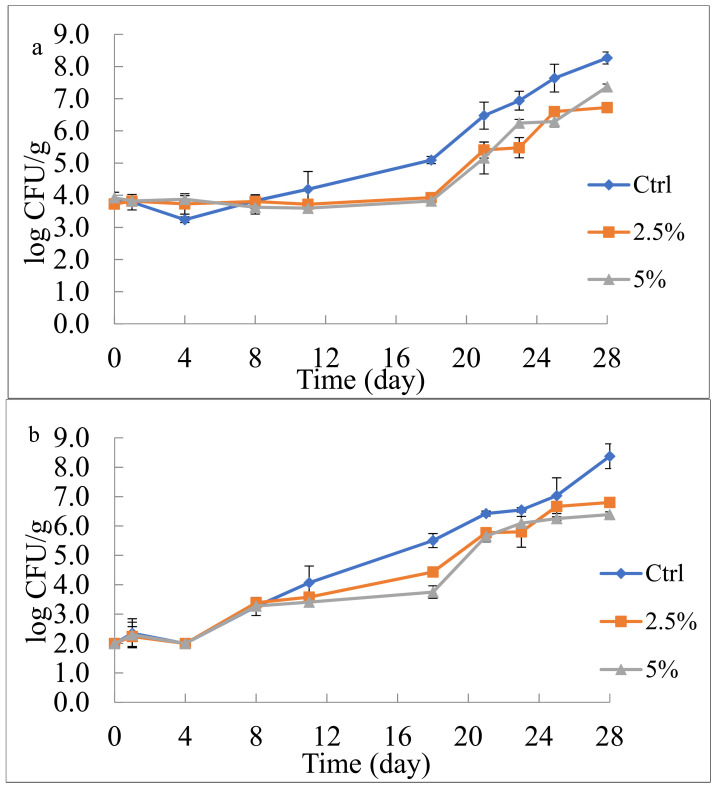
Evolution of total mesophilic (**a**) and psychrotrophic (**b**) bacteria in fruit salad during 28 days of refrigerated storage (5 °C). Ctrl: fruit salad without pomegranate peel powder; 2.5%: fruit salad with 2.5% (*w*/*v*) pomegranate peel powder; 5%: fruit salad with 5% (*w*/*v*) pomegranate peel powder.

In particular, the evolution of total mesophilic and psychrotrophic bacterial counts is shown in Figure 1ab. As can be seen, for the total mesophilic bacteria (Figure 1a), the initial microbial concentration of both the control and active fruit salads was around 3.80 log CFU/g. During the first 8 days, the Ctrl and active samples maintained, more or less, the initial microbial concentration; in the following days, a marked difference appeared between them. Specifically, the Ctrl sample showed a significant microbial increase up to 8.27 log CFU/g after 28 days, whereas, both salads with peel powder maintained the same microbial count for more than 2 weeks. After this long lag phase, the bacteria gradually grew; however, the increase in the viable cell concentration of active samples was less pronounced than that recorded for the control sample. The control salad reached 8 log CFU/g, whereas both active systems remained around 7 log CFU/g. Most probably, the pomegranate peel powder, rich in active compounds, inhibited microbial growth during the first stage of storage, and subsequently, microorganisms, accustomed to the conditions, began to grow [17,35]. Sun et al. [23] suggested that the antimicrobial activity of pomegranate peels is related to the combined effect of polyphenols, sterols, and pentacyclic triterpenoid compounds.

For the total psychrotrophic bacteria, a trend similar to the total mesophilic count was observed (Figure 1b). During the exponential phase, and also at the stationary phase, in the active sample, the microbial load was lower than that observed in the control sample. In fact, also in this case, control salad reached 8 log CFU/g, whereas, the active samples remained between 6 and 7 log CFU/g after the 4 weeks of observation. These antimicrobial effects on different microbial groups are not surprising because it is well recognized from studies reported in the literature that the bioactive substances contained in pomegranate peel can inhibit growth of different microbial species [15,21,27].

Looking at recorded data, product acceptability in terms of microbial quality can be defined. According to the French Regulation, fresh-cut fruit remained acceptable until the total count of mesophilic and psychrotrophic bacteria reaches 5 × 10^7^ CFU/g [36]. Considering the results of the control and active systems reported in Figure 1 1a,b, it can be inferred that the control salad remained acceptable for about 24 days, whereas, both salads with pomegranate peel powder were found below the microbial threshold for the entire observation period (28 days). Therefore, according to these experimental findings, the lowest concentration of pomegranate by-product is enough to assure a longer microbial stability of fresh-cut nectarine and pineapple mix than the control salad.

While slight differences in pH values between the control and active systems were found, no differences in pH were recorded between the fruit and its fructose syrup (data not shown). To give a more precise idea about the product pH, after 28 days of storage the pH values observed for the active samples were about 3.72 and 3.68 for the 2.5 and 5% samples, respectively, whereas, a pH of about 4.46 was recorded for the control sample.

**Figure 2:**
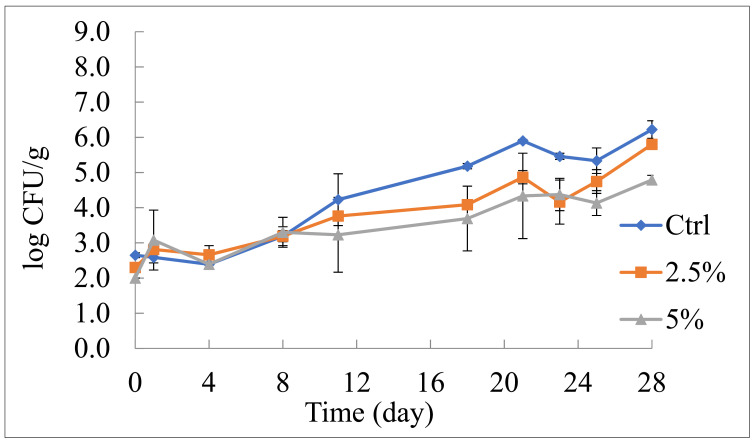
Evolution of yeasts in fruit salad during 28 days of refrigerated storage (5 °C). Ctrl: fruit salad without pomegranate peel powder; 2.5%: fruit salad with 2.5% (*w*/*v*) pomegranate peel powder; 5%: fruit salad with 5% (*w*/*v*) pomegranate peel powder.

Regarding the effects of the by-products on the other monitored spoilage groups, it is worth noting that the peel powder added to fruit salad significantly affected yeast growth, as shown in Figure 2. Although the initial microbial concentration of the active samples was slightly lower than the control, the trend of the three investigated samples was similar during the first 8 days. After this period, yeast growth rate changed. In particular, a more rapid increase was found in the control system and delayed kinetics were recorded for both active packages. A final count of 6.22 log CFU/g was measured in the Ctrl sample, whereas, 5.80 and 4.79 log CFU/g were found in the active samples with 2.5 and 5% pomegranate peel powder, respectively. The fruit salad with the highest concentration of pomegranate peel was the least contaminated at the end of the storage period, thus confirming the antimicrobial activity of this by-product against fungal spoiling [16]. Gull et al. [26] also observed that chitosan coating enriched with pomegranate peel extract was effective in protecting apricot from yeast spoiling when stored for 30 days.

**Figure 3:**
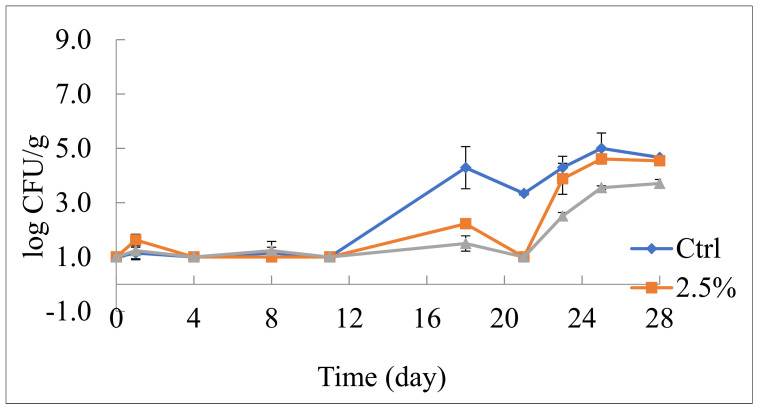
Evolution of lactic acid bacteria in fruit salad during 28 days of refrigerated storage (5 °C). Ctrl: fruit salad without pomegranate peel powder; 2.5%: fruit salad with 2.5% (*w*/*v*) pomegranate peel powder; 5%: fruit salad with 5% (*w*/*v*) pomegranate peel powder.

In relation to lactic acid bacteria (LAB), the effects of the peel powder were very marked. It is also striking to observe that between the two concentrations of by-product, the highest one was the most effective (Figure 3). In particular, in the control sample, LAB remained low for one week and then increased up to about 5 log CFU/g. In both active salads, LAB had a long lag phase with a very small microbial load for more than two weeks. The cells then grew; however, the viable cell concentration reached the value of about 5 log CFU/g in the sample with 2.5% peel powder and about 4 log CFU/g in the sample with the highest peel powder concentration.

**Figure 4:**
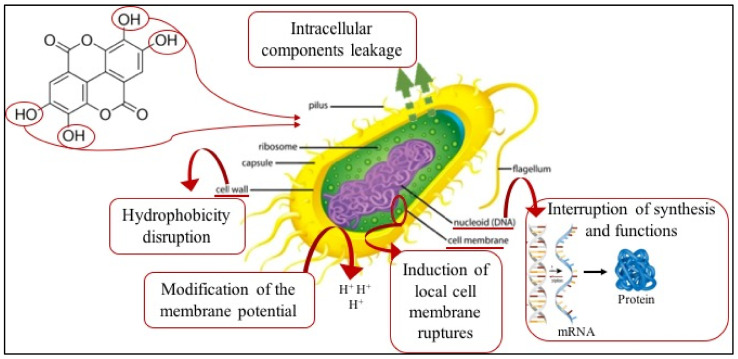
Mechanism of action of polyphenols on a microbial cell.

Polyphenols contained in the active powder can justify the observed antimicrobial activity [35,37]. One of the factors that can influence the efficacy of these compounds against different microbial and fungal groups is the position of the hydroxyl groups (OH) in the aromatic ring of polyphenols. Hydroxyl groups can interact with microbial cell membranes to make them more permeable and cause the loss of cellular components, as well as damage microbial metabolic processes [35]. A representation of the complex mechanisms involved in the antimicrobial effects of polyphenols is also provided in Figure 4.

Regarding mold proliferation, in all packages a concentration of about 3 log CFU/g was measured, without any statistically significant differences among samples (data not shown). With high probability, the washing of fruit in chlorinated water before cutting and the refrigerated storage conditions controlled the mold proliferation [10].

No significant differences were also found in the viable cell concentration of *Enterobacteriaceae*, which recorded a final concentration of about 4.5 log CFU/g in all investigated samples (data not shown). This finding about *Enterobacteria* means that general hygienic conditions were adopted during production and processing.

### 3.2. Sensory Quality Decay during Refrigerated Storage

**Figure 5:**
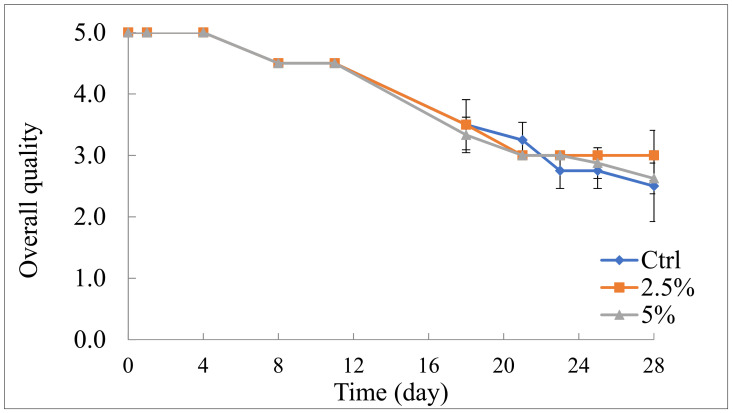
Overall quality of fruit salad during 28 days of refrigerated storage (5°C). Ctrl: fruit salad without pomegranate peel powder; 2.5%: fruit salad with 2.5% (*w*/*v*) pomegranate peel powder; 5%: fruit salad with 5% (*w*/*v*) pomegranate peel powder.

With regard to the effects of pomegranate peel on product acceptability, the changes in sensory quality during storage are reported in Figure 5. As one would expect, during the entire storage period, a gradual decrease in the sensory quality was found in both the Ctrl and active samples [33]. As can be inferred from the data shown in the figure, fruit salads, regardless of the type of sample, remained acceptable for around 3 weeks. Then, defects appeared that made the product disagreeable to panelists. Table 1 lists the specific sensory attribute scores as assessed by the panel. The data highlight that the main attribute responsible for product sensory deterioration is the odor; texture and general appearance also contributed to product rejection. This finding is not surprising because microbial and fungal proliferation, water loss, and enzymatic reactions occurring during fruit storage generally also cause changes in the product quality, primarily in terms of odor and color [2,38]. Loss of firmness can be correlated with tissue degradation [4,5]. Therefore, the trends shown in Figure 4 for fruit salads’ overall quality effectively reflect the decrease in odor, appearance, and texture that occurred in all of the samples.

When comparing the fruit salad sensory quality of the investigated samples, it appears that pomegranate peel powder did not worsen the main sensory fruit salad attributes. On the contrary, the panelists appreciated a slight herbaceous smell and a marked fruity aroma in the active samples that were stored for about 20 days.

The enhancement in sensory quality was also recorded when pomegranate peel powder was added to pancake formulations [30] and when the same by-product was adopted as a breading for fresh cod sticks [31].

In the current study, the overall quality decay of the investigated samples reported in Figure 5 highlights that the degradation of the control sample after 3 weeks was very rapid. On the contrary, in the active samples the sensory quality decay was slower. The reasons for the abovementioned difference among samples are strictly linked to the fact that the control sample becomes unacceptable because of the degradation of the whole product, whereas the active samples were mainly refused for the turbidity of the syrup in the containers, which negatively affected the visual quality of the fruit salad. This drawback represents one of the main problems faced when by-products are applied to food and necessitates further research to find valid solutions to boost by-product recycling [32]. The inclusion of peel powder in either an edible or biodegradable polymeric matrix could be a valid strategy [14].

## 4. Conclusions

Fresh-cut fruits, although providing health benefits and convenience to consumers, are highly perishable. This study proves that traditional non-edible parts of pomegranate fruit, such as the peel, can be used to slow down the detrimental phenomena responsible for fruit salad unacceptability. In particular, the current research investigated the effects of 2.5 and 5% pomegranate peel powder placed on the bottom of a fruit salad container filled with fructose syrup. The investigated by-product exerted good antimicrobial and antifungal activity; in fact, it prolonged the lag phase and reduced the cell viable concentration at the stationary phase of total mesophilic and psychrotrophic bacteria, yeasts, and lactic acid bacteria, primarily when the highest peel powder concentrations were used. Consequently, it is possible to conclude that fruit salads with 2.5 and 5% of pomegranate peel powder were less contaminated than the control sample. The effects of peel powder are further prized if the sensory quality of fruit is considered. In fact, the investigated by-product did not compromise fruit acceptability; moreover, similar sensory quality decay kinetics were recorded in the control and active samples. Further studies are needed to solve the problem of suspended powder in fructose syrup because this flaw can make the product unattractive. Therefore, considering the positive impacts by-product valorization has on both the economy and the environment, we have another study in progress to optimize the addition of this valuable peel powder to make the final product more appealing.

## Figures and Tables

**Table 1:** Sensory attributes of active and non-active fresh fruit salad during 28 days of storage at 5 °C.

Samples	Storage Time (Days)										
	0	1	4		8	11	18	21	23	25	28
Appearance	Control	5.0 ± 0.0 ^a^	5.0 ± 0.0 ^a^	5.0 ± 0.0 ^a^	4.5 ± 0.0 ^a^	4.5 ± 0.0 ^b^	3.5 ± 0.5 ^a^	3.4 ± 0.3 ^b^	3.3 ± 0.3 ^a^	3.4 ± 0.3 ^b^	2.9 ± 0.3 ^a^
	2.5%	5.0 ± 0.0 ^a^	5.0 ± 0.0 ^a^	5.0 ± 0.0 ^a^	4.5 ± 0.5 ^a^	3.9 ± 0.3 ^a^	3.2 ± 0.3 ^a^	3.0 ± 0.0 ^a^	3.0 ± 0.0 ^a^	3.0 ± 0.0 ^a,b^	2.9 ± 0.3 ^a^
	5%	5.0 ± 0.0 ^a^	5.0 ± 0.0 ^a^	5.0 ± 0.0 ^a^	4.2 ± 0.3 ^a^	3.9 ± 0.3 ^a^	3.2 ± 0.3 ^a^	3.0 ± 0.0 ^a^	3.0 ± 0.0 ^a^	2.9 ± 0.3 ^a^	3.0 ± 0.0 ^a^
Odor	Control	5.0 ± 0.0 ^a^	5.0 ± 0.0 ^a^	5.0 ± 0.0 ^a^	4.5 ± 0.0 ^a^	4.0 ± 0.0 ^a^	3.8 ± 0.3 ^a^	3.2 ± 0.3 ^a^	2.8 ± 0.3 ^a^	2.9 ± 0.3 ^a^	2.3 ± 0.5 ^a^
	2.5%	5.0 ± 0.0 ^a^	5.0 ± 0.0 ^a^	5.0 ± 0.0 ^a^	5.0 ± 0.0 ^b^	4.5 ± 0.0 ^b^	3.9 ± 0.3 ^a^	2.9 ± 0.3 ^a^	2.8 ± 0.3 ^a^	3.0 ± 0.0 ^a^	3.0 ± 0.3 ^a^
	5%	5.0 ± 0.0 ^a^	5.0 ± 0.0 ^a^	5.0 ± 0.0 ^a^	4.5 ± 0.0 ^a^	4.5 ± 0.0 ^b^	3.5 ± 0.0 ^a^	2.9 ± 0.3 ^a^	2.9 ± 0.3 ^a^	2.9 ± 0.3 ^a^	2.7 ± 0.3 ^a^
Texture	Control	5.0 ± 0.0 ^a^	5.0 ± 0.0 ^a^	5.0 ± 0.0 ^a^	4.5 ± 0.0 ^a^	4.5 ± 0.0 ^a^	3.5 ± 0.0 ^a^	3.3 ± 0.3 ^a^	3.5 ± 0.0 ^a^	2.9 ± 0.3 ^a^	2.9 ± 0.3 ^a^
	2.5%	5.0 ± 0.0 ^a^	5.0 ± 0.0 ^a^	5.0 ± 0.0 ^a^	4.5 ± 0.0 ^a^	4.5 ± 0.0 ^a^	3.9 ± 0.3 ^a^	3.5 ± 0.0 ^a^	3.3 ± 0.3 ^a^	3.3 ± 0.3 ^a^	3.0 ± 0.3 ^a^
	5%	5.0 ± 0.0 ^a^	5.0 ± 0.0 ^a^	5.0 ± 0.0 ^a^	4.5 ± 0.0 ^a^	4.5 ± 0.0 ^a^	3.9 ± 0.3 ^a^	3.5 ± 0.0 ^a^	3.4 ± 0.3 ^a^	3.2 ± 0.3 ^a^	3.0 ± 0.0 ^a^

For each sensory attribute, data (±SD, *n* = 2) marked with different superscript letters (^a,b^) in each column are significantly different (*p* < 0.05). Control: fruit salad without pomegranate peel powder; 2.5%: fruit salad with 2.5% (*w*/*v*) pomegranate peel powder; 5%: fruit salad with 5% (*w*/*v*) pomegranate peel powder.

## Data Availability

The raw data will be made available upon request.
